# High-precision U-Pb data and reference age for Emerald Lake apatite

**DOI:** 10.1016/j.dib.2025.111464

**Published:** 2025-03-24

**Authors:** Francisco E. Apen, Sean P. Gaynor, Blair Schoene

**Affiliations:** a Geosciences Department, Princeton University, Princeton, NJ 08544, United States; b School of Earth and Sustainability, Northern Arizona University, Flagstaff, AZ 86011, United States; c United States Geological Survey, Geology, Geophysics & Geochemistry Science Center, Denver, CO 80225, United States

**Keywords:** U-Pb isotopes, Geochronology, Apatite, Thermal ionization mass spectrometry

## Abstract

New isotope dilution thermal ionization mass spectrometry U-Pb data for Emerald Lake apatite demonstrate its potential as a reference material for geochronology. A three-dimensional ^238^U/^206^Pb-^207^Pb/^206^Pb-^204^Pb/^206^Pb isochron produces a 95.2 ± 1.1 Ma date with an initial Pb isotopic composition of ^206^Pb/^204^Pb = 18.85 ± 0.19 and ^207^Pb/^204^Pb = 15.68 ± 0.10 (n = 5, MSWD = 9.5). These data yield a weighted mean initial Pb-corrected ^206^Pb/^238^U date of 95.18 ± 0.10 Ma (n = 5, MSWD = 1.5) and a weighted mean initial Pb-corrected ^207^Pb/^235^U date of 95.20 ± 0.17 Ma (n = 5, MSWD = 0.5). The new high-precision U-Pb age of Emerald Lake apatite further enables its utility as a reference material for *in situ* U-Pb apatite geochronology. Aliquots of Emerald Lake apatite are available for distribution for use in future studies.

Specifications Table

**Table:** 

Subject	
Specific subject area	High precision U-Pb isotopic data for Emerald Lake apatite collected by isotope dilution thermal ionization mass spectrometry (ID-TIMS).
Type of data	Table, Figure, Image, Analyzed, Processed
Data collection	An apatite crystal was cut and crushed, and fragments were picked, cleaned, dissolved, spiked with a U-Pb tracer solution, and processed using dilute HBr- and HCl-based anion exchange chromatography. U and Pb isotopes were measured separately on an IsotopX Phoenix TIMS at Princeton University. The U-Pb data and uncertainties for each measurement were calculated using ET_Redux (version 3.7.0) and plotted using the IsoplotR software package (version 6.5).
Data source location	Specimens come from the Emerald Lake pluton, Yukon, Canada, and were procured from an online vendor. Data collected at the Department of Geosciences, Princeton University (USA) and are stored at the School of Earth and Sustainability, Northern Arizona University (USA).
Data accessibility	Repository name: Mendeley Data Data identification number: DOI:10.17632/34jn7b8m6z.2 Direct URL to data: https://data.mendeley.com/datasets/34jn7b8m6z/2
Related research article	None.

## 1 Value of the Data

• U-Pb isotope data that are collected using microbeam methods are calibrated against matrix-matched reference materials. Homogeneous and well-characterized reference materials are essential for ensuring precise and accurate age determinations with these methods.
• New high-precision isotope dilution thermal ionization mass spectrometry U-Pb data from natural apatite from Emerald Lake, Canada, produce uniform dates and demonstrate its potential for use as a reference material for U-Pb isotope analyses.
• Aliquots of Emerald Lake apatite are available for distribution to the broader geochronological community to facilitate inter-laboratory assessment of quality and reproducibility of U-Pb data.

## 2 Background

Microbeam methods, such as laser ablation inductively coupled plasma mass spectrometry (LA-ICP-MS) and secondary ionization mass spectrometry (SIMS), enable rapid, high spatial-resolution measurements of U-Pb isotopes and subsequent age determinations of minerals. The relatively high throughput afforded by these methods is particularly advantageous for isochron age determinations, as the precision of an isochron age depends on the number and compositional range of measurements used to populate an isochron, in addition to the precision of individual analyses [1]. These analytical techniques require the use of well-characterized and homogeneous matrix-matched reference materials for calibration and age normalization, which also ensure accurate and reproducible results by accounting for instrumental drift and matrix effects during analysis (e.g., [2]).

Apatite [Ca_5_(PO_4_)_3_(OH,F,Cl)] is a phosphate mineral that is common in a variety of rock types. It readily incorporates U and Th, making it useful as a geochronometer, although it also incorporates non-radiogenic (“common”) Pb during crystallization. Consequently, age determinations of apatite are primarily calculated from isochrons, due to the necessity to correct for the composition of the common Pb. Isotopic analyses of apatite by LA-ICP-MS and SIMS have proliferated, and a variety of reference materials have been developed for such purposes (e.g., [[3], [4], [5], [6], [7]]). Establishing reference materials for U-Pb geochronology requires isotopic composition measurements to be independently verified, primarily using isotope dilution thermal ionization mass spectrometry (ID-TIMS), which avoids matrix effects inherent in microbeam methods and offers higher precision and accuracy isotopic and age determinations (e.g., [3,4,6,7]). New ID-TIMS U-Pb data for Emerald Lake apatite are presented to allow for its use as a reference material for apatite geochronology.

Chew et al. [8] first suggested apatite from the Emerald Lake pluton (Yukon, Canada) as a reference material for apatite U-Pb geochronology. The Emerald Lake pluton is a composite Cretaceous intrusion, ranging from augite syenite to biotite granite in composition [9], and samples from the granitic phase are what has been suggested for use for reference apatite. Using LA-ICP-MS, Chew et al. [8] obtained a ^238^U/^206^Pb-^207^Pb/^206^Pb isochron date of 92.5 ± 3.3 Ma (n = 33, MSWD = 1.6) based on a regression anchored to a Stacey and Kramers [10] model initial Pb composition. An independent age of Emerald Lake apatite has not been previously established and has been assumed to be 92.2 ± 0.9 Ma based on a single analysis of a multi-grain titanite U-Pb aliquot reported from the same locality [9]. We directly date Emerald Lake apatite using ID-TIMS.

## 3 Data Description

**Fig. 1:**
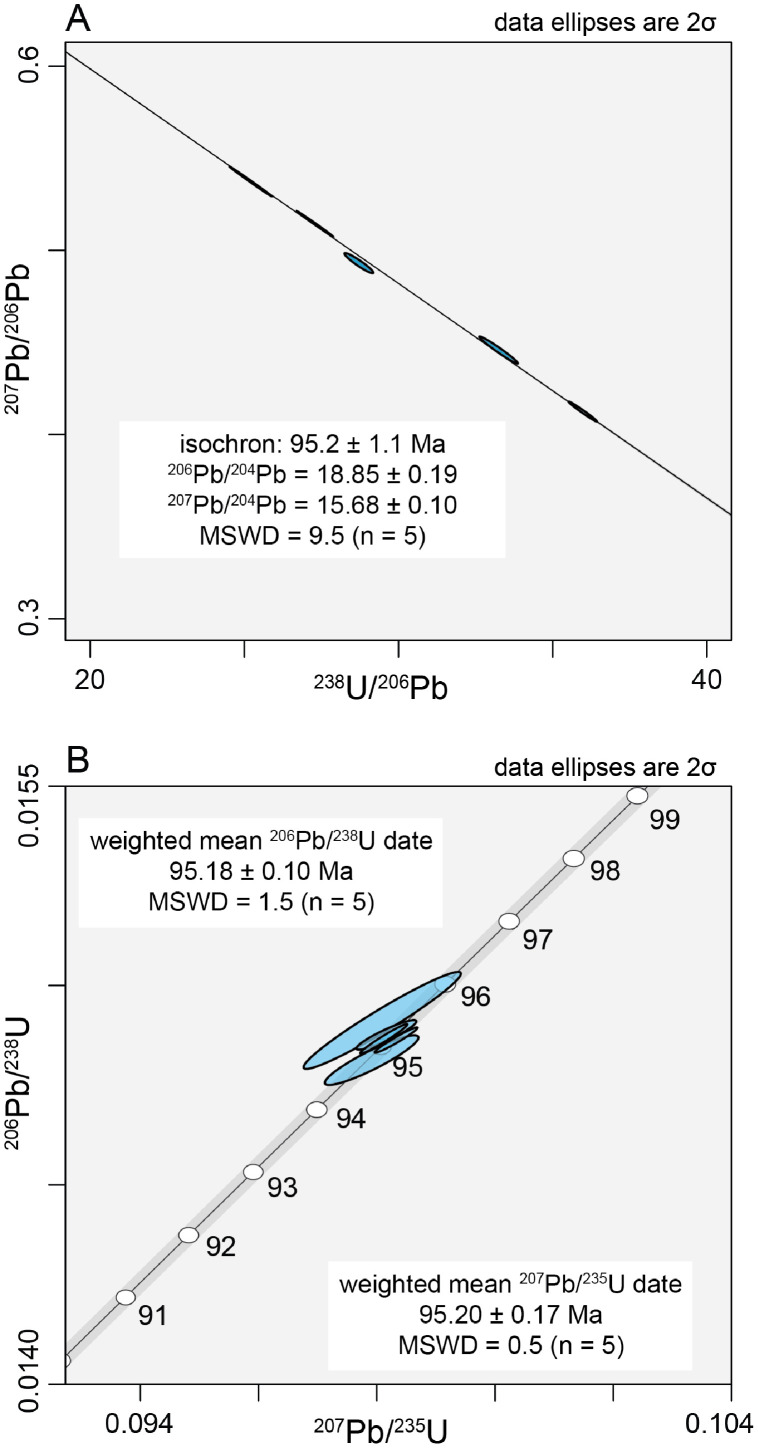
A) Tera-Wasserburg concordia diagram for initial Pb-uncorrected U-Pb data for Emerald Lake apatite. B) Wetherhill concordia diagram for initial Pb-corrected Emerald Lake apatite. The data are corrected for initial Pb based on the three-dimensional isochron regression.

The analyzed apatite specimens were originally collected between 1994 and 1996 from miarolitic cavities within the Emerald Lake pluton. Seven U-Pb isotopic analyses of an Emerald Lake apatite crystal were undertaken by ID-TIMS. Two analyses yielded relatively high uncertainty due to poor Pb ionization and were therefore omitted from the dataset. A three-dimensional ^238^U/^206^Pb-^207^Pb/^206^Pb-^204^Pb/^206^Pb isochron date of 95.2 ± 1.1 Ma was obtained for Emerald Lake apatite, with an initial Pb isotopic composition of ^206^Pb/^204^Pb = 18.85 ± 0.19 and ^207^Pb/^204^Pb = 15.68 ± 0.10 (all 2σ) (n = 5, mean squared weighted deviation [MSWD] = 9.5) (Fig. 1A). Using the initial Pb composition from the three-dimensional isochron regression to correct for common Pb, five analyses overlapped concordia and produced a weighted mean ^206^Pb/^238^U date of 95.18 ± 0.10 Ma (MSWD = 1.5) and a weighted mean ^207^Pb/^235^U date of 95.20 ± 0.17 Ma (MSWD = 0.5) (Fig. 1B). The new high-precision ID-TIMS U-Pb age for Emerald Lake apatite provides a robust benchmark by which data produced by future microbeam studies can be evaluated.

## 4 Experimental Design, Materials and Methods

**Fig. 2:**
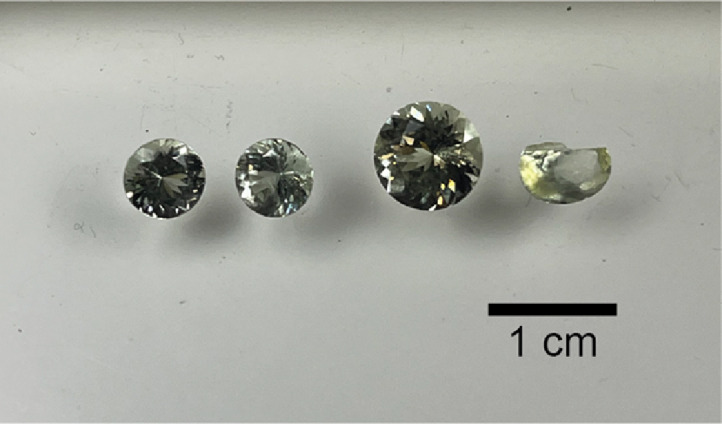
Image of Emerald Lake apatite. Fragments of these materials are available for distribution for use as reference materials. Note the right-hand apatite was cut in half for processing for ID-TIMS analysis.

Four untreated, centimeter-size, gem-quality apatite crystals from the Emerald Lake pluton were procured from an online vendor (Fig. 2). Analytical procedures follow those outlined in [7] and are summarized herein. One crystal was sawn in half and one piece from that material was crushed using a ceramic mortar and pestle. Seven milligram-scale fragments were hand-picked and subsequently rinsed three times in high-purity water. These fragments were then transferred into individual 200 μL Savillex microcapsules with added 6 M HCl and a mixed ^205^Pb-^233^U-^235^U tracer solution (ET535) [11,12] and left to dissolve at 210°C for 12 h in a Parr bomb. Following dissolution, the resulting solutions were dried to salts on a hotplate and then redissolved in 1 M HBr. Separation of Pb and U from the dissolved material was done using dilute HBr- and HCl-based anion exchange chromatography modified from [13]. The chemically purified Pb and U aliquots were dried down with added trace H_3_PO_4_ and were loaded separately onto outgassed zone-refined Re filaments with silicic acid activator [14].

Isotopes of U and Pb were analyzed separately using an IsotopX Phoenix TIMS at Princeton University. Lead isotopes were measured in dynamic mode using a Daly photomultiplier, and U isotope ratios were measured as UO_2_ in static mode using Faraday collectors coupled to 10^12^ Ω resistor amplifiers. Mass fractionation of Pb was calculated from previous repeat measurements of samples spiked with the ET2535 tracer solution [11], which resulted in a value of α = 0.182 ± 0.041 % a.m.u (2σ). Uranium isotope ratios were corrected for mass fractionation using the ^233^U/^235^U composition of the spike [11], assuming a sample ^238^U/^235^U of 137.818 ± 0.045 (2σ) [15]. Interferences in UO_2_ analyses were corrected in-run using ^18^O/^16^O ratios calculated using intensity on mass 269 [16]. All analyses were corrected for laboratory blanks, which were estimated to contribute 0.36 ± 0.15 pg of Pb during the HBr- and HCl-based anion exchange chromatography, based on 15 repeat measurements of total procedural blanks performed during span of this study. The Pb blank composition has ^206^Pb/^204^Pb = 19.15 ± 0.54, ^207^Pb/^204^Pb = 16.00 ± 0.38 and ^208^Pb/^204^Pb = 39.02 ± 3.60 (2σ). The final U-Pb data and associated uncertainties were calculated using the algorithms of [17]. The data were plotted and isochrons calculated using the IsoplotR software package [18]. These data were reported using the community accepted distinctions between dates and ages (e.g., [[2], [19]]). Analyses of Earthtime ET100Ma standard solution performed during the period of these analyses resulted in a weighted mean ^206^Pb/^238^U date of 100.1633 ± 0.0093 Ma (MSWD = 0.93; n = 17), which is consistent with the inter-laboratory calibrated value of 100.173 ± 0.003 Ma for the ET100Ma standard solution reported recently [19]. The complete ID-TIMS data set is reported in Table 1 and is also available in an online data repository [20].

**Table 1:** ID-TIMS data for Emerald Lake apatite.

(a) a1, a2 etc. are labels for single apatite fragments.

(b) Model Th/U ratio iteratively calculated from the radiogenic 208Pb/206Pb ratio and 206Pb/238U age.

(c) Pb* and Pbc represent radiogenic and common Pb, respectively; mol % ^206^Pb* with respect to radiogenic, blank and initial common Pb.

(d) Measured ratio corrected for spike and fractionation only. Fractionation estimated at 0.18 +/- 0.03 %/a.m.u. for Daly analyses, based on analysis of NBS-981 and NBS-982.

(e) Corrected for fractionation, spike, and common Pb; up to 1 pg of common Pb was assumed to be procedural blank: 206Pb/204Pb = 19.15 ± 0.27; 207Pb/204Pb = 16.00 ± 0.19; 208Pb/204Pb = 39.02 ± 1.80 (all uncertainties 1-sigma).

Excess over blank was assigned to initial common Pb, based on the initial Pb intercept of a 3-D isochron regression (206Pb/204Pb = 18.85 ± 0.19 and 207Pb/204Pb = 15.68 ± 0.10)

(f) Errors are 2-sigma, propagated using the algorithms of Schmitz and Schoene (2007).

(g) Calculations are based on the decay constants of Jaffey et al. (1971). 206Pb/238U and 207Pb/206Pb ages corrected for initial disequilibrium in 230Th/238U using Th/U [magma] = 3.

(h) Corrected for fractionation, spike, and blank Pb only.

(i) Nominal fraction weights estimated from photomicrographic grain dimensions, adjusted for partial dissolution during chemical abrasion.

(j) Nominal U and total Pb concentrations subject to uncertainty in photomicrographic estimation of weight and partial dissolution during chemical abrasion.

## Limitations

Aliquots of the Emerald Lake reference apatite are available from the authors (Fig. 2). As with all natural reference materials, the original analyzed material is available in limited quantities. Additional apatite crystals, assumed to be of the same age as the analyzed crystal, are also available for distribution.

## Ethics Statement

The authors declare that they have read and follow the ethical requirements for publication in Data in Brief and confirm that the current work does not involves human subjects, animal experiments, or any data collected from social media platforms.

## CRediT authorship contribution statement

**Francisco E. Apen:** Conceptualization, Investigation, Formal analysis, Data curation, Writing – original draft, Visualization, Funding acquisition. **Sean P. Gaynor:** Methodology, Validation, Investigation, Formal analysis, Writing – review & editing. **Blair Schoene:** Methodology, Validation, Writing – review & editing, Supervision, Funding acquisition.

## Data Availability

(Mendeley Data).ID-TIMS U-Pb data for Emerald Lake apatite (Original data)
